# Accelerated Activation of SOCE Current in Myotubes from Two Mouse Models of Anesthetic- and Heat-Induced Sudden Death

**DOI:** 10.1371/journal.pone.0077633

**Published:** 2013-10-15

**Authors:** Viktor Yarotskyy, Feliciano Protasi, Robert T. Dirksen

**Affiliations:** 1 Department of Physiology and Pharmacology, University of Rochester Medical Center, Rochester, New York, United States of America; 2 Center for Research on Ageing & Department of Neuroscience and Imaging, Università Gabriele d'Annunzio, Chieti, Italy; SUNY College of Nanoscale Science and Engineering, United States of America

## Abstract

Store-operated calcium entry (SOCE) channels play an important role in Ca^2+^ signaling. Recently, excessive SOCE was proposed to play a central role in the pathogenesis of malignant hyperthermia (MH), a pharmacogenic disorder of skeletal muscle. We tested this hypothesis by characterizing SOCE current (I_SkCRAC_) magnitude, voltage dependence, and rate of activation in myotubes derived from two mouse models of anesthetic- and heat-induced sudden death: 1) type 1 ryanodine receptor (RyR1) knock-in mice (Y524S/+) and 2) calsequestrin 1 and 2 double knock-out (dCasq-null) mice. I_SkCRAC_ voltage dependence and magnitude at -80 mV were not significantly different in myotubes derived from wild type (WT), Y524S/+ and dCasq-null mice. However, the rate of I_SkCRAC_ activation upon repetitive depolarization was significantly faster at room temperature in myotubes from Y524S/+ and dCasq-null mice. In addition, the maximum rate of I_SkCRAC_ activation in dCasq-null myotubes was also faster than WT at more physiological temperatures (35-37°C). Azumolene (50 µM), a more water-soluble analog of dantrolene that is used to reverse MH crises, failed to alter I_SkCRAC_ density or rate of activation. Together, these results indicate that while an increased rate of I_SkCRAC_ activation is a common characteristic of myotubes derived from Y524S/+ and dCasq-null mice and that the protective effects of azumolene are not due to a direct inhibition of SOCE channels.

## Introduction

Malignant hyperthermia (MH) is a pharmacogenetic disorder triggered by exposure to halogenated anesthetics (e.g. halothane) and depolarizing skeletal muscle relaxants (e.g. succinylcholine) [1]. MH episodes are characterized by a dramatic rise in core body temperature, respiratory acidosis, skeletal muscle rigidity, hypermetabolism, rhabdomyolysis, hyperkalemia, and cardiac arrhythmia [1]. MH attacks are lethal if not reversed rapidly by removal of triggering agent, cooling the patient, and administration of dantrolene, the only FDA-approved antidote for an MH crisis [2]. Interestingly, some MH susceptible individuals have experienced similar life-threatening non-anesthetic hypermetabolic reactions upon exposure to heat stress (hot temperatures and high humidity), strenuous exercise, or febrile illness [3-9]. 

 MH crises result from uncontrolled Ca^2+^ release from the sarcoplasmic reticulum (SR) that ultimately leads to a dramatic and sustained rise in intracellular Ca^2+^ [1]. MH in humans usually results from missense mutations in the type I ryanodine receptor (RyR1), which functions as the Ca^2+^ release channel in the sarcoplasmic reticulum (SR) of skeletal muscle [1]. Recently, multiple mouse models for heat- and halothane-induced sudden death were developed including knock-in of mutations in RyR1 linked to MH in humans [10,11] and knockout of calsequestrin1 (Casq1) [12,13], the primary SR Ca^2+^ binding protein in skeletal muscle. The Y524S mutation increases RyR1 Ca^2+^ leak and susceptibility to activation. Y524S knock-in mice exhibit anesthetic- and heat-induced sudden death and are an established mouse model of MH [10,14]. On the other hand, Casq1 deficiency results in both reduced SR Ca^2+^ storage and loss of Casq1-mediated regulation of RyR1 Ca^2+^ release [12,15]. Like Y524S mice, Casq1-null mice also exhibit anesthetic- and heat-induced sudden death, but mutations in the CASQ1 gene have not been linked to MH in humans [12,13].

Recent evidence suggests that SR Ca^2+^ depletion due to uncontrolled Ca^2+^ release during an MH episode activates store-operated calcium entry (SOCE), which exacerbates Ca^2+^ overload and hypermetabolism during an MH crisis. Indeed, early studies conducted during the development of the *in vitro* caffeine and halothane contracture test, the gold standard for MH diagnosis in North America, revealed that this test fails when conducted using Ca^2+^-free extracellular solutions [16-19]. More recently, increased SOCE activation during halothane-induced Ca^2+^ release was demonstrated in mechanically skinned human skeletal muscle fibers from MH susceptible patients [20]. Indirect measures of SOCE have suggested an inverse relationship between the level of Casq1 expression and the magnitude of SOCE in myotubes [21] and adult muscle fibers [22]. In addition, SOCE was reduced by preincubation of myotubes and muscle fibers with azumolene, a more water-soluble dantrolene analog [22,23]. 

Together, these studies provide provocative evidence for a central pathogenic role of SOCE in the intracellular Ca^2+^ overload in skeletal muscle that occurs during an MH crisis. However, direct measurements of SOCE channel activity and sensitivity to block by dantrolene/azumolene in skeletal muscle cells from an established MH mouse model have not been tested. Therefore, we used a whole-cell voltage clamp technique described previously [24] to characterize the magnitude, voltage dependence, and activation rate of the SOCE current in myotubes (termed I_SkCRAC_) derived from *Ryr1* ^*Y524S/+*^ (Y524S/+) knock-in mice. We also conducted parallel experiments in homozygous Casq1 and Casq2 double knock-out (dCasq-null) mice that also exhibit anesthetic- and heat-induced sudden death. Finally, we determined the effect of temperature and azumolene on I_SkCRAC_ amplitude and activation in myotubes from wild-type, Y524S/+, and dCasq-null mice. The results demonstrate that while SOCE channel activation is faster in Y524S/+, and dCasq-null myotubes and azumolene does not act by directly blocking the SOCE channel current. 

## Matherials and Methods

### Ethical approval

All animals were housed in a pathogen-free area at the University of Rochester and all experiments were carried out in accordance with procedures reviewed and approved by the University of Rochester University Committee on Animal Resources (UCAR). 

### Primary myotube cultures

Myotube cultures were prepared from newborn control wild-type (WT) C57Bl6, heterozygous *Ryr1* ^*Y524S/+*^ knock-in (Y524S/+), and homozygous Casq1/2 double knock-out (dCasq-null) mice as previously described [25]. Since mouse myotubes express both the Casq1 and Casq2 isoforms [26], experiments were conducted in myotubes derived from dCasq-null mice in order to assess the impact of Casq deficiency on I_SkCRAC_ properties. Mice used to generate myotube cultures were genotyped by PCR analysis of tail snip genomic DNA using previously established protocols [10,27]. All electrophysiological experiments were performed on individual myotubes from 8-11 day old cultures. 

### Whole-cell patch clamp recordings

The whole-cell patch-clamp technique was used to isolate I_SkCRAC_ in myotubes using an approach described previously [24]. Briefly, myotubes were bathed in an external recording solution containing (in mM): 138 TEA-Methanesulfonate, 10 CaCl_2_, 10 HEPES, 1 MgCl_2_, 0.1 nifedipine, pH7.4 adjusted with TEA-OH. Low resistance patch pipettes (resistance in the bath ranged from 0.5 - 1.2 Mohm) were filled with an internal solution comprised of (in mM): 140 Cs-Methanesulfonate, 10 HEPES, 20 Na_2_-EGTA, 4 MgCl_2_, pH7.4 adjusted with CsOH. Upon membrane rupture and establishment of whole cell mode, myotubes were voltage clamped at a holding potential of -80 mV and then repetitive ramp depolarizations were delivered every 2 s. Series resistance after compensation (80%) was always <2 Mohms. Each ramp depolarization consisted of a 1 s step to 0 mV (to trigger depolarization-induced Ca^2+^ release and inactivate Na^+^ and T-type Ca^2+^ channels) followed by a 30 ms step to +40 mV (to further inactivate sodium channels and monitor outward leak current), a 200 ms ramp from +100 mV to -100 mV, and a final 65 ms step to -80 mV.

 Whole-cell currents were recorded with an Axopatch 200A amplifier (Molecular Devices, Sunnyvale, CA) and filtered at 2 kHz using an inline four-pole Bessel filter. Data were digitized at 8.3 kHz using a DigiData 1200 interface (Molecular Devices, Sunnyvale, CA). Capacitative currents were minimized (>90%) using the capacitative transient cancellation feature of the amplifier. Cell capacitance (C_m_) was determined prior to capacitance cancelation by integration of the capacity transient resulting from a +10-mV pulse applied from the holding potential and used to compare I_SkCRAC_ current density (pA/pF) obtained from myotubes of different size. Data were collected at room temperature (21-23°C, RT) and physiological temperature (35–37°C, PT) in WT control, Y524S/+, and dCasq-null myotubes. 

### Off-line data analyses

All data analyses were performed using Igor Pro 6 (Wavemetrics, Lake Oswego, OR) and Clampfit10 (Molecular Devices, Sunnyvale, CA) software as described previously [24]. I_SkCRAC_ amplitude was determined by averaging the inward current during 20 ms of the final voltage step to -80 mV at the end of each I_SkCRAC_ voltage protocol. The time course of I_SkCRAC_ activation was determined by plotting I_SkCRAC_ amplitude for each depolarization protocol delivered every 2 seconds. I_SkCRAC_ voltage dependence was obtained from individual 200 ms voltage ramp sweeps (1 mV/ms) elicited during each voltage protocol. Ramp currents obtained before I_SkCRAC_ activation or the first ramp current elicited upon establishing whole-cell mode were used to subtract linear leak and capacitative currents from sweeps during I_SkCRAC_ activation. The time required to reach 10% (T10%), 50% (T50%), and 90% (T90%) of maximal I_SkCRAC_ during repetitive depolarization was used as an index of the speed of SOCE channel activation. The maximum rate of SOCE channel activation was determined from the peak of the differential for the time course of normalized I_SkCRAC_ amplitude (dI_Norm_/dt). All data are presented as mean ± standard error of mean. Statistical significance (p < 0.05) was determined using a Student's two-tailed *t*-test. One-way analysis of variance and Tukey’s HSD *post hoc* test was used to test for differences among three or more independent groups. 

## Results

### I_SkCRAC_ activation is accelerated in myotubes from Y524S/+ and dCasq-null mice

**Figure 1:**
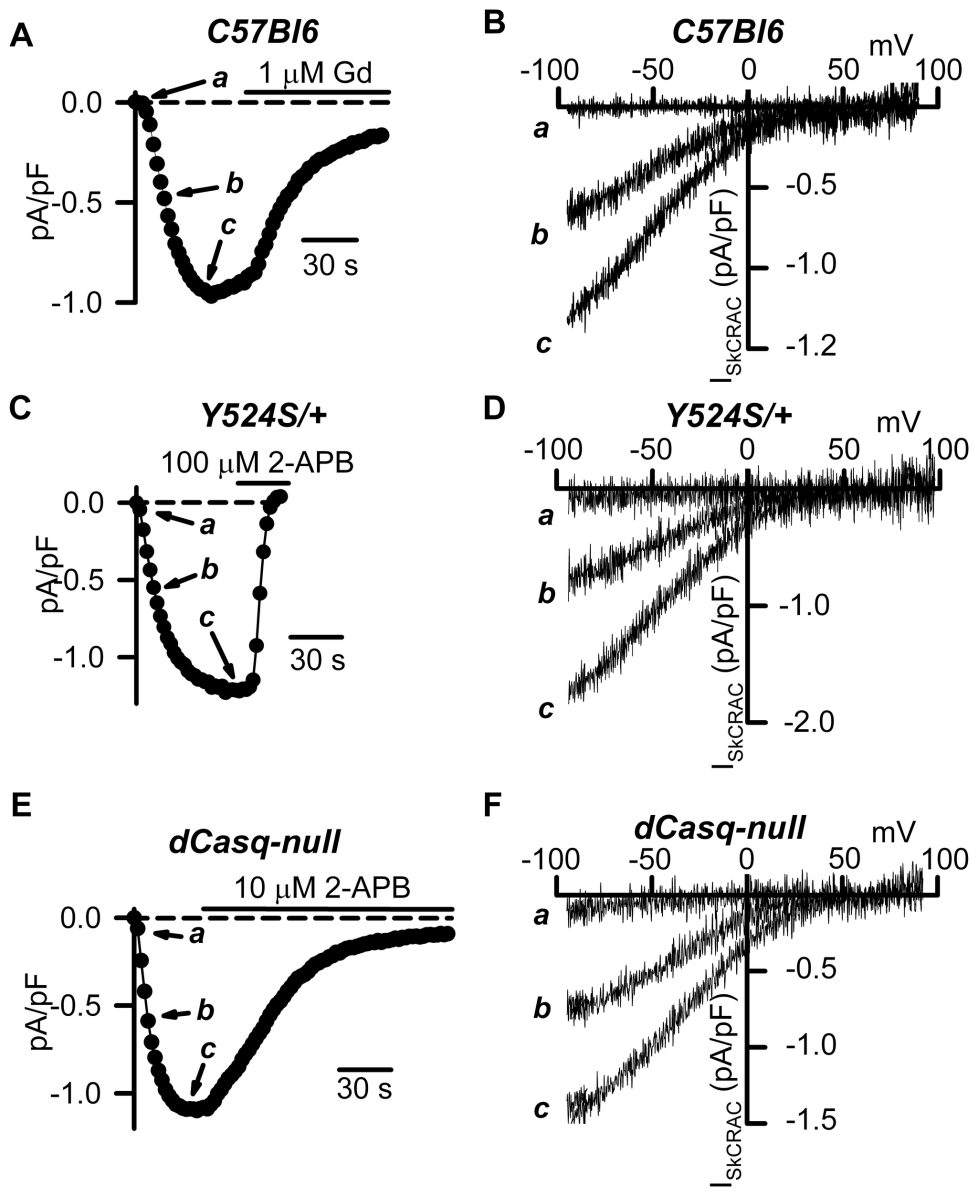
I_SkCRAC_ properties in myotubes from Y524S/+ and dCasq-null mice. (**A**, **C**, and **E**) Representative time courses for I_SkCRAC_ activation during repetitive ramp depolarization in the presence of 20 mM internal EGTA in myotubes from WT C57Bl6 (**A**), Y524S/+ (**C**), and dCasq-null (**E**) mice. I_SkCRAC_ was measured at -80 mV and plotted against time. Following full activation, I_SkCRAC_ was blocked by addition of either 1 µM Gd^2+^ (**A**), 100 µM 2-APB (**C**), or 10 µM 2-APB (**E**). Arrows indicate time points a, b, and c used to illustrate I_SkCRAC_ voltage dependence in panels **B**, **D** and **F**. Dashed lines represent the zero current level and solid lines indicate drug application. (**B**, **D**, and **F**) Voltage dependence of I_SkCRAC_ for the representative WT C57Bl6 (**B**), dCasq-null (**D**), and Y524S/+ (**F**) myotubes shown in **A** **C**, and **E**, respectively.

**Figure 2:**
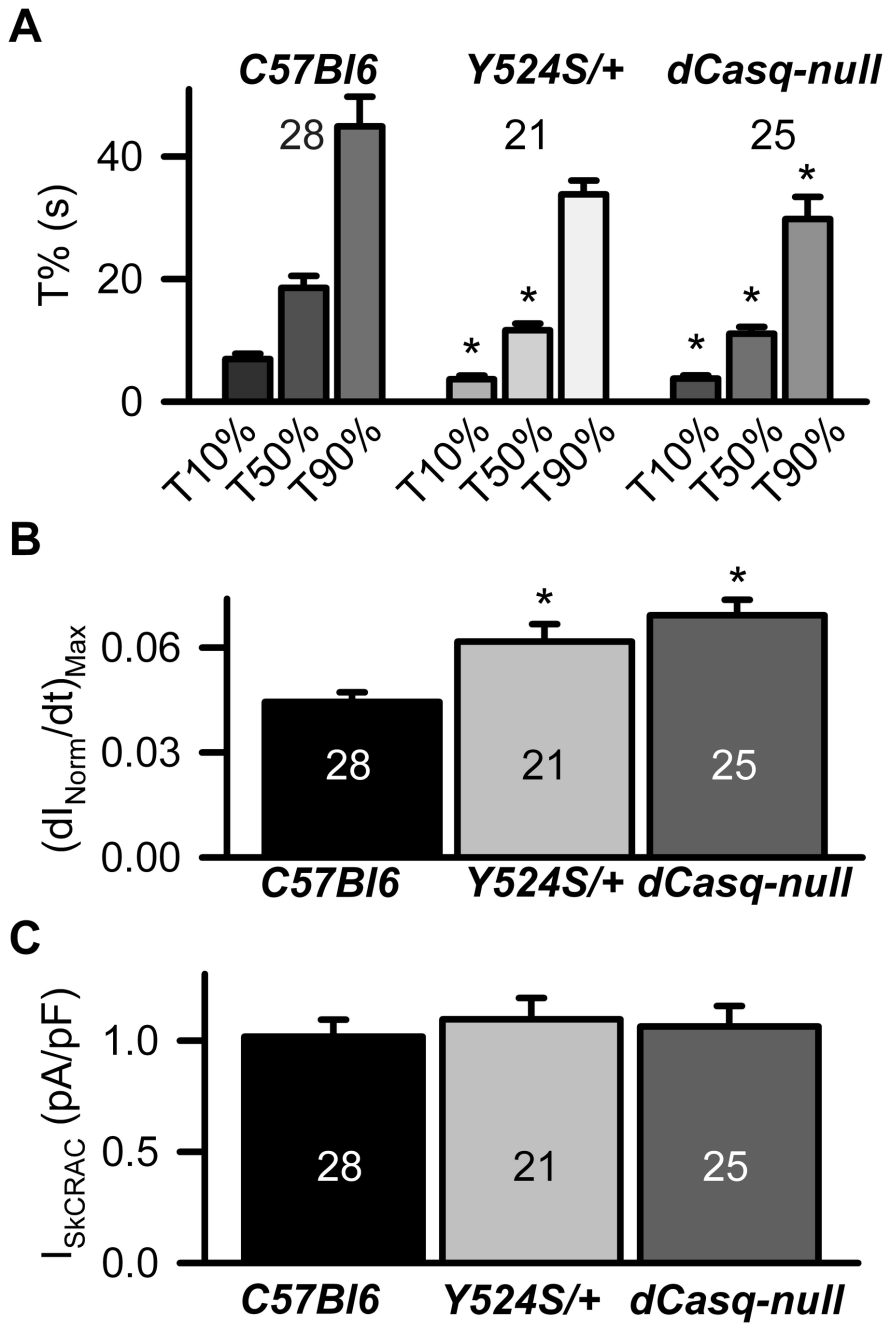
Rate of I_SkCRAC_ activation is increased in myotubes from Y524S/+ and dCasq-null mice. (**A**) Average (±SE) time required for 10% (T10%), 50% (T50%), and 90% (T90%) activation of I_SkCRAC_ is significantly increased in myotubes from Y524S/+ and dCasq-null mice. *Denotes statistically significant differences compared to WT C57Bl6 (p<0.05). (**B**) Average (±SE) maximum rate of I_SkCRAC_ activation is increased in myotubes from Y524S/+ and dCasq-null mice. *Denotes statistically significant differences compared to WT C57Bl6 (p<0.05). (**C**) Average (±SE) peak I_SkCRAC_ current density recorded at -80 mV was not different between myotubes from WT C57Bl6, Y524S/+ and dCasq-null mice.

 We previously described a whole-cell voltage clamp approach to directly quantify the magnitude, voltage dependence, and rate of SOCE current (I_SkCRAC_) activation in skeletal myotubes [24]. These studies demonstrated that RyR1-dependent SR Ca^2+^ depletion governs the rate of I_SkCRAC_ activation during repetitive depolarization. Thus, we hypothesized that the enhanced RyR1 sensitivity to activation and increased SR Ca^2+^ “leak” in myotubes from Y524S/+ mice would result in an increased rate of I_SkCRAC_ activation during repetitive depolarization. To test this prediction, we monitored I_SkCRAC_ activation in myotubes from control WT (C57Bl6) and Y524S/+ mice during repetitive ramp depolarization delivered at 0.5 Hz recorded under conditions in which SR Ca^2+^ reuptake was inhibited by inclusion of a high intracellular concentration of a strong Ca^2+^ buffer (20 mM EGTA) (Figures 1 A and C). Similar to that observed in WT myotubes [24], I_SkCRAC_ in Y524S/+ myotubes exhibited strong inward rectification (Figure 1 B) and was inhibited by both 100 µM 2-APB (103 ± 3 %, n = 7) (Figure 1 C and Figure S1 B in File S1) and 1 µM Gd^3+^ (66.9 ± 3.4%, n = 7) ( Figure S1 A in File S1). The time course of I_SkCRAC_ activation during repetitive depolarization was significantly faster in Y524S/+ myotubes (Figure 2A and B). Specifically, the time required for 10% and 50% full activation of I_SkCRAC_ during repetitive depolarization (T10% and T50%, respectively) were significantly reduced in myotubes from Y524S/+ mice compared to that from WT mice (Figure 2A, Table S1 in File S1). In addition, also the maximum rate of I_SkCRAC_ activation ([dI_Norm_/dt]_Max_), calculated from the peak of the first derivative of the normalized I_SkCRAC_ activation time course, was significantly increased in myotubes from Y524S/+ mice (Figure 2 B).

 We also hypothesized that a similar increase in the rate of I_SkCRAC_ activation would be observed in Casq-null myotubes since Casq deficiency should result in faster store depletion due to the combined effects of reduced SR Ca^2+^ storage and loss of Casq-dependent RyR1 inhibition [12,15]. Therefore, we also determined the impact of Casq deficiency on I_SkCRAC_ magnitude, voltage-dependence, and rate of activation. Since mouse myotubes express both type 1 and type 2 Casq isoforms [26], we compared I_SkCRAC_ magnitude, voltage dependence, and rate of activation in whole-cell voltage clamp experiments of myotubes derived from control WT (C57Bl6) and homozygous dCasq-null [15,27,28] mice (Figures 1 and 2). Similar to that observed for myotubes from Y524S/+ mice, I_SkCRAC_ in dCasq-null myotubes activated rapidly during repetitive depolarization (Figure 1 E), exhibited strong inward rectification (Figure 1 F), and was inhibited by both 1 µM Gd^3+^ (59.1 ± 3.9%, n=7) (Figure S1 A in File S1) and 10 µM 2-APB (94.7 ± 8.9%, n = 5) (Figure 1 E and Figure S1 B in File S1). In addition, the rate of I_SkCRAC_ activation during repetitive depolarization was accelerated in dCasq-null myotubes (Figure 2 A and B). Specifically, T10%, T50%, and T90%were all significantly reduced for dCasq-null myotubes. On average, T10%, T50%, and T90% were reduced on 46%, 40% and 34%, respectively (Figure 2 A, Table S1 in File S1). In addition, dI_Norm_/dt_Max_ was also significantly increased in myotubes from dCasq-null mice (Figure 2 B). In spite of the increased rate of I_SkCRAC_ activation in myotubes from Y524S/+ and Casq-null mice, average (±SE) peak I_SkCRAC_ current density at -80 mV was not different between WT (1.02 ± 0.08 pA/pF, n=28), Y524S/+ (1.10 ± 0.10 pA/pF, n = 21), and dCasq-null (1.06 ± 0.09 pA/pF, n=25) myotubes (Figure 2 C), indicating that maximal SOCE channel activity was not altered by either the Y524S mutation in RyR1 or Casq deficiency***.***

### Temperature accelerates I_SkCRAC_ activation in myotubes from Y524S/+ and dCasq-null mice

**Figure 3:**
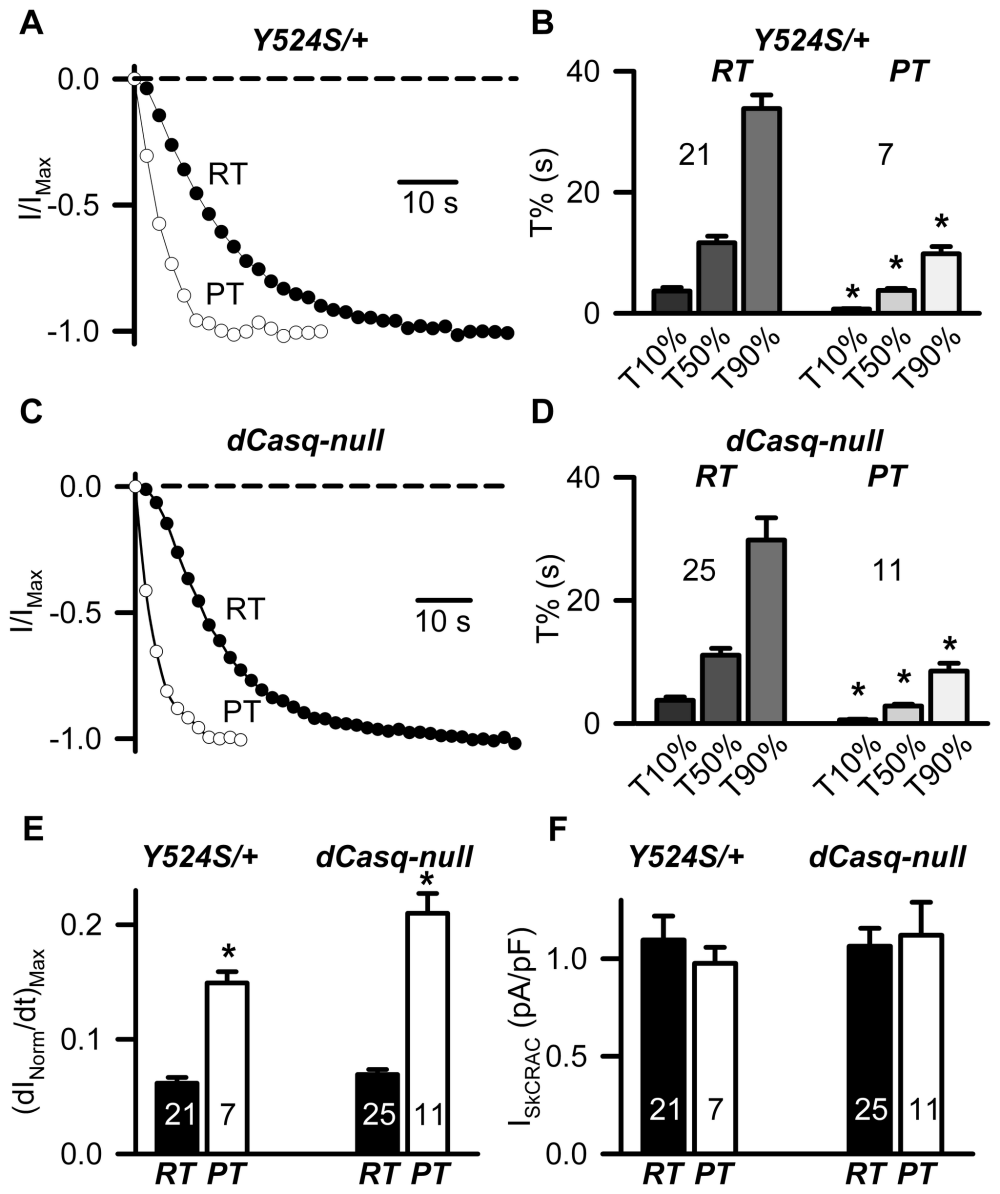
Rate of I_SkCRAC_ activation is accelerated at physiological temperature in myotubes from Y524S/+ and dCasq-null mice. (**A**) Representative time courses for activation of normalized I_SkCRAC_ (I/I_Max_) recorded from Y524S/+ myotubes at either room temperature (RT, *filled* *circles*) or physiological temperature (PT, *open* *circles*). The dashed line represents the zero current level. (**B**) Average (±SE) time required for 10% (T10%), 50% (T50%), and 90% (T90%) I_SkCRAC_ activation in Y524S/+ myotubes at RT (left) and PT (right). *Denotes statistically significant differences compared to RT (p<0.05). (**C**) Representative time courses for activation of normalized I_SkCRAC_ (I/I_Max_) recorded from dCasq-null myotubes at either RT (filled circles) or PT (open circles). The dashed line represents the zero current level. (**D**) Average (±SE) time required for 10% (T10%), 50% (T50%), and 90% (T90%) activation of I_SkCRAC_ in dCasq-null myotubes at RT (left) and PT (right). (**E**) Average (±SE) maximum rate of I_SkCRAC_ activation at RT (filled bars) or PT (open bars) determined from the peak of the first derivative of the I/I_max_ time course ((dI_Norm_/dt)_max_) in Y524S/+ (left) and dCasq-null (right) myotubes. *Denotes statistically significant differences compared to RT (p<0.05). (**F**) Average (±SE) peak I_SkCRAC_ current density recorded at -80 mV at RT (filled bars) or PT (open bars) in Y524S/+ (left) and dCasq-null (right) myotubes.

Our previous study demonstrated that I_SkCRAC_ activation in control myotubes is increased at physiological temperatures [24]. Therefore, we tested if rate of I_SkCRAC_ activation in Y524S/ + and dCasq-null myotubes was also further increased at physiological temperature 35–37°C (PT). As observed for control myotubes, the rate of I_SkCRAC_ activation in Y524S/+ myotubes was also faster at PT (Figure 3 A and B). For the representative experiment shown in Fig. 3 A, T50% was reached only 11.2 s after initiation of the repetitive ramp depolarization protocol, while a similar level of I_SkCRAC_ activation took >3-fold longer to develop at RT. On average, T10%, T50%, and T90% were reduced by 81.0%, 67.7%, and 70.9%, respectively at PT (Figure 3 B, Tables S1 and S2 in File S1). Similarly, I_SkCRAC_ activation was also significantly faster at physiological temperature (Figure 3 C) in dCasq-null myotubes, with average T10%, T50%, and T90% being reduced 85%, 75%, and 71%, respectively (Figure 3 D and Tables S1 and S2 in File S1). Consistent with these results, the average maximal rate of I_SkCRAC_ activation (dI_Norm_/dt)_Max_ was significantly increased at PT in myotubes from both Y524S/+ and dCasq-null mice (Figure 3 E), though peak steady-state I_SkCRAC_ current density was not different between RT and PT in either myotube genotype (Figure 3 F).

The rates of I_SkCRAC_ activation at PT in myotubes derived from WT, Y524S/+, and dCasq-null mice were statistically evaluated by ANOVA analysis (control results taken from [24]), although no statistically significant differences were observed for either T10%, T50%, or T90% (Figure S2 A in File S1). However, the maximum rate of I_SkCRAC_ activation (dI_Norm_/dt)_Max_ was significantly increased in myotubes from dCasq-null mice (Figure S2 B in File S1). No differences were observed in either peak I_SkCRAC_ current density ( Figure S2 C in File S1) or voltage dependence (data not shown). 

### Azumolene pretreatment does not alter I_SkCRAC_ magnitude or rate of activation

**Figure 4:**
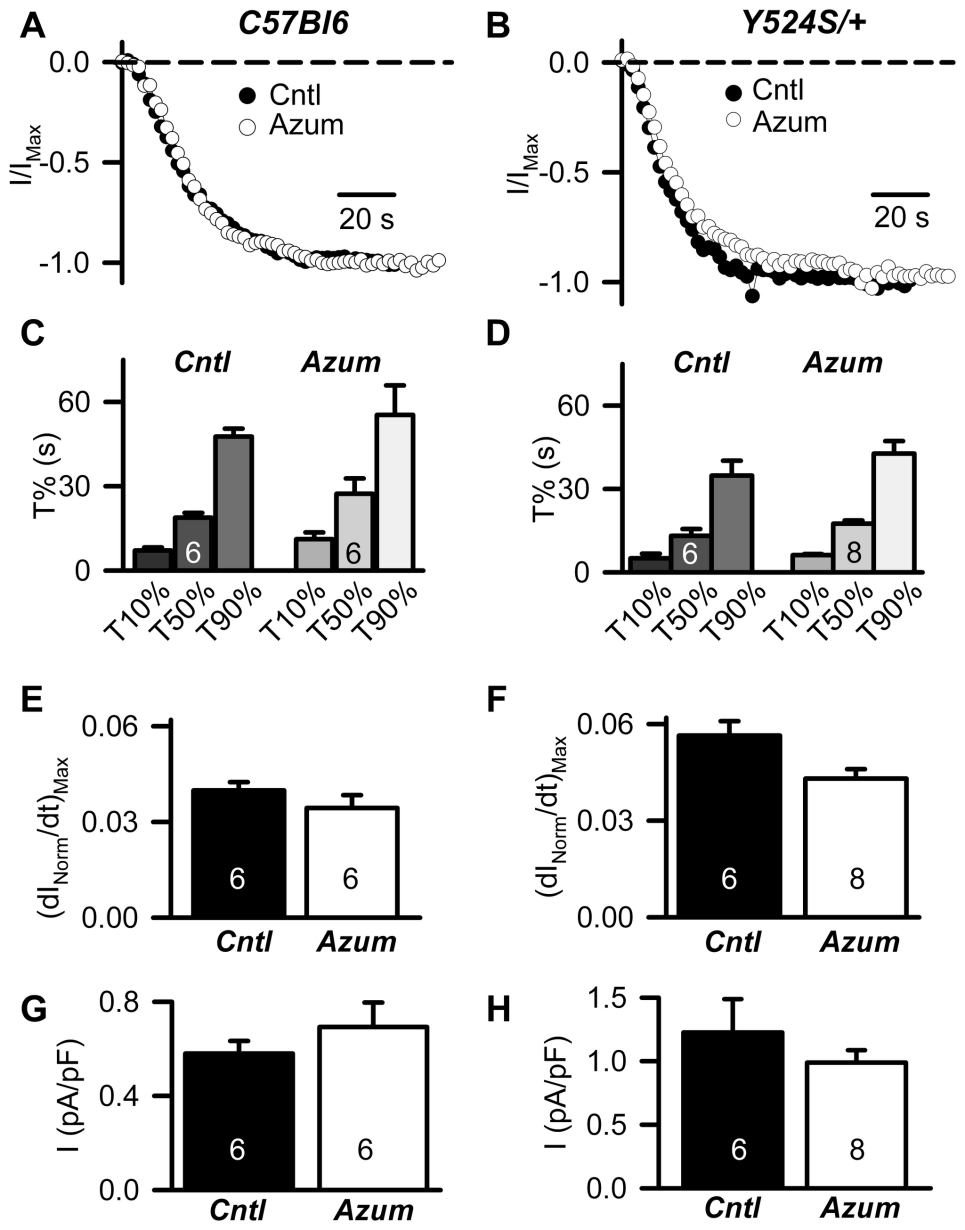
Azumolene pretreatment does not alter I_SCRAC_ magnitude or rate of activation in myotubes from WT C57Bl6 and Y524S/+ mice. (**A** and **B**) Representative time courses for activation of normalized I_SkCRAC_ (I/I_Max_) recorded from WT C57Bl6 (**A**) and Y524S/+ (**B**) myotubes in the absence (filled circles) and presence (open circles) or 50 µM azumolene (Azum). (**C** and **D**) Average (±SE) time required for 10% (T10%), 50% (T50%), and 90% (T90%) activation of I_SkCRAC_ in WT C57Bl6 (**C**) and Y524S/+ (**D**) myotubes in the absence (left) and presence (right) or 50 µM azumolene. (**E** and **F**) Average (±SE) maximum rate of I_SkCRAC_ activation determined from the peak of the first derivative of the I/I_max_ time course ((dI_Norm_/dt)_max_) in WT C57Bl6 (**E**) and Y524S/+ (**F**) myotubes in the absence (left) and presence (right) or 50 µM azumolene. (**G** and **H**) Average (±SE) peak I_SkCRAC_ current density recorded at -80 mV in WT C57Bl6 (**G**) and Y524S/+ (**H**) myotubes in the absence (left) and presence (right) or 50 µM azumolene. All experiments were conducted at room temperature.

 Previous studies reported that pretreatment of myotubes and adult muscle fibers with 20 µM azumolene inhibits a component of SOCE coupled to RyR1 activation [23]. However, since these studies employed only indirect measures of SOCE channel function (e.g. Mn^2+^ quench, reduction in Rhod-2 fluorescence trapped within sealed transverse tubules), potential direct effects of azumolene on SOCE channel function remain unclear. Therefore, we determined the effect of azumolene pretreatment on I_SkCRAC_ magnitude and rate of activation in whole-cell voltage clamp experiments of WT and Y524S/+ myotubes (Figure 4). Myotubes were first pretreated with either vehicle control or 50 µM azumolene for 1 min prior to whole-cell voltage clamping and then maintained in this solution throughout activation of I_SkCRAC_ as performed in Figures 1-3. The time course of I_SkCRAC_ activation was not different in the presence or absence of 50 µM azumolene in myotubes from both WT (Figure 4 A) and Y524S/+ (Figure 4 B) mice. Consistent with the results shown in Figure 1, the rate of I_SkCRAC_ activation was faster in Y524S/+ myotubes compared to that of WT myotubes (Figures 4 C-F, Table S3 in File S1). However, pretreatment with 50 µM azumolene did not significantly alter T10%, T50%, or T90% activation of I_SkCRAC_ in myotubes from either WT (Figure 4 C) or Y524S/+ mice (Figure 4 D). Azumolene pretreatment also failed to significantly alter either the maximum rate of I_SkCRAC_ activation (Figures 4 E and F) or maximal steady-state I_SkCRAC_ current density (Figure 4 G and H). In addition, I_SkCRAC_ amplitude was not altered either following washout of pre-applied azumolene or when azumolene was added after I_SkCRAC_ was fully activated (data not shown). Finally, azumolene pretreatment also failed to significantly alter maximum steady-state I_SkCRAC_ amplitude in RyR1-null myotubes (control: 0.74 ± 1.4 pA/pF, n = 5; azumolene: 1.08 ± 0.17 pA/pF, n = 8) or T10%, T50%, and T90%. As a positive control for drug activity, voltage clamp studies found that the maximum rate of depolarization-induced calcium release was significantly reduced (15.1 ± 3.4%, n = 5; p < 0.05) within 1 minute after addition of 25 µM azumolene. Together, these results indicate that azumolene does not directly inhibit SOCE channels activated during repetitive depolarization.

## Discussion

 In this study, we used voltage clamp experiments to directly measure SOCE channel activity (i.e. I_SkCRAC_ magnitude, voltage dependence, pharmacology, and rate of activation) in myotubes from two different mouse models of heat- and halothane-induced sudden death (i.e. Y524S/+ and dCasq-null mice). We found that rate of I_SkCRAC_ activation during repetitive depolarization at room temperatures was accelerated in both Y524S/+ and dCasq-null myotubes in the absence of a change in current magnitude or voltage dependence. While the rate of I_SkCRAC_ activation was increased at PT for all genotypes, the maximum rate of I_SkCRAC_ activation at PT was significantly faster than WT only for myotubes derived from dCasq-null mice. Finally, we found that azumolene, a more water-soluble dantrolene analogue, did not significantly alter either I_SkCRAC_ magnitude or rate of activation. These results indicate that while myotubes from MH mice exhibit an increased susceptibility for activation of SOCE during repetitive depolarization, the SOCE channel in myotubes is not a direct molecular target of azumolene.

### Increased rate of I_SkCRAC_ activation in mouse models of anesthetic/heat-induced sudden death

 We found that the rate of I_SkCRAC_ activation at RT during repetitive depolarization was significantly increased in the absence of a change in maximal SOCE current density in myotubes derived from Y524S/+ and dCasq-null mice, two well-established mouse models of heat- and anesthetic-induced sudden death [10,12,29]. On the other hand, a statistically significant increase in the maximum rate of I_SkCRAC_ activation at PT was resolved for dCasq-null myotubes, but not for myotubes from both Y524S/+ mice. Given the relatively rapid rate of I_SkCRAC_ activation observed at 37°C even in WT myotubes (50% activation in ~3 sec while I_SkCRAC_ is monitored only every 2 sec), our ability to resolve a further increase in activation at the higher temperature is limited. As a result, the data obtained at RT represents a more reliable reflection of the difference in rate of I_SkCRAC_ activation between myotubes derived from WT mice and those from mice that exhibit halothane- and heat-induced sudden death.

 This observed increase in the rate of I_SkCRAC_ activation in Y524S/+ and dCasq-null myotubes likely reflects a more rapid rate of SR Ca^2+^ store depletion during repetitive depolarization as a result of either reduced SR Ca^2+^ storage capacity (dCasq-null myotubes) or enhanced RyR1 Ca^2+^ leak (Y524S/+ mytoubes). Our observation that the rate of I_SkCRAC_ activation is increased in dCasq-null myotubes is consistent with prior reports that Casq-deficiency results in a significant reduction in the time required to achieve store depletion (and thus, I_SkCRAC_ activation) during both sustained [30] and repetitive [15] depolarization. Since core body temperature can rise up to 43°C (>109°F) during heat stress or anesthetic-induced lethal episodes [2], an increased susceptibility for temperature-dependent store depletion and I_SkCRAC_ activation would provide strong positive feedback whereby an increase in temperature promotes deeper SR Ca^2+^ depletion and subsequent enhanced Ca^2+^ entry through SOCE channels to drive additional Ca^2+^ entry, uncontrolled muscle contraction, and heat generation. As ATP reserves become used up, SERCA pumps would be unable to adequately refill SR Ca^2+^ stores, contractile filaments would be unable to relax, and persistent SOCE channel activation would permit massive Ca^2+^ influx from a near infinite extracellular Ca^2+^ source. For this reason, inhibition of SOCE channels may represent a novel molecular target to interrupt this destructive positive feedback loop, and thus, prevent and/or rescue patients from deadly heat- and anesthetic-induced MH episodes. However, direct evidence for an increased rate of I_SkCRAC_ activation in muscle during lethal anesthetic-induced MH events in humans will require further investigation. 

### Mechanism of temperature-dependent SOCE channel activation

 Since a marked increase in core temperature is a fundamental component of MH crises, we determined the temperature dependence of I_SkCRAC_ magnitude and rate of activation during repetitive depolarization. We found that while maximal I_SkCRAC_ current density is similar at RT and PT, the rate of I_SkCRAC_ activation is significantly faster at PT in myotubes from WT control [24], Y524S/+, and dCasq-null mice (Figure 3). The underlying mechanism for the increase in the rate of I_SkCRAC_ activation at PT is unclear, but could theoretically result from a temperature-dependent increase in the rate of SR Ca^2+^ depletion, STIM1 multimerization [31], and/or STIM1/Orai1 coupling [24]. However, the effects of temperature on I_SkCRAC_ activation are not likely to be due to increased STIM1 multimerization or coupling to Orai1 since near immediate (<1s) SOCE activation occurs even at room temperature upon rapid store depletion [32,33]. Also, the temperature-dependent increase in the rate of SR Ca^2+^ depletion is apparently not influenced by Casq1-mediated Ca^2+^ buffering or regulation of RyR1 activity since a similar ~3-fold increase in I_SkCRAC_ activation rate was observed in myotubes from WT and dCasq-null mice. Thus, we propose that faster I_SkCRAC_ activation at PT most likely reflects an increased rate of SR Ca^2+^ depletion during repetitive depolarization. This idea is consistent with previous observations that L-type Ca^2+^ currents and voltage-gated SR Ca^2+^ release exhibit faster kinetics and are activated at more negative voltages [29] at PT. In addition, steady-state “window” RyR1 Ca^2+^ release exhibits a hyperpolarizing shift in voltage-dependence at elevated temperatures [14]. Moreover, the kinetics of ryanodine binding to SR vesicles, a surrogate for RyR channel activation, is also increased at PT [29]. Together, these results indicate that RyR1-dependent Ca^2+^ release, and thus, the rate of SR Ca^2+^ depletion and subsequent I_SkCRAC_ activation, is increased at PT. Given the increase in core temperature that develops during an MH event, the temperature dependence of I_SkCRAC_ activation would be expected to exacerbate myoplasmic Ca^2+^ overload. However, additional studies are needed to quantify the temperature dependence of SR Ca^2+^ depletion and I_SkCRAC_ activation contributes to the myoplasmic Ca^2+^ overload uncontrolled muscle contraction, and heat generation that occurs during an MH crisis. 

### The role of calsequestrin in SOCE channel regulation

An emerging body of evidence supports the importance of SOCE in skeletal muscle physiology and pathology [21,23,32,34-37]. The role of Casq in retrograde regulation of SOCE in skeletal muscle was first suggested by Ma and colleagues [21-23], where a reciprocal relationship between Casq expression and SOCE function was demonstrated. Specifically, overexpression of Casq1 in myotubes inhibited SOCE [21], while SOCE function was increased following Casq1 knockdown in adult muscle fibers [22]. However, what is unclear from these experiments is whether Casq reduces SOCE channel function by retrograde inhibition of SOCE channel number and/or open probability or by simply making it more difficult for RyR1 to deplete SR Ca^2+^ content since Casq markedly increases the local Ca^2+^ buffer capacity near sites of RyR1 Ca^2+^ release ([38]). The direct measurements of I_SkCRAC_ magnitude and rate of activation reported here support the later interpretation since Casq deficiency accelerated SOCE channel activation in the absence of a significant effect on maximum I_SkCRAC_ current density. This conclusion is further supported by recent findings that SR Ca^2+^ store depletion during repetitive stimulation occurs more rapidly in muscle fibers from Casq1-deficient mice than from WT mice and that the rate required for refilling these stores correlates with stimulation frequency and the degree of SR Ca^2+^ depletion, consistent with SOCE channel activation [15]. 

Using direct electrophysiological measurements of I_SkCRAC_ activity, we found that SOCE channel activation during repetitive depolarization is significantly accelerated in myotubes from dCasq-null mice. This increase in the rate of I_SkCRAC_ activation in dCasq-null myotubes is consistent with Casq deficiency reducing SR Ca^2+^ buffer capacity near sites of RyR1 Ca^2+^ release, thus making it easier for Ca^2+^ release during repetitive depolarization to promote SR Ca^2+^ depletion. These findings are also consistent with the enhanced “SR evacuability” (or ability of the SR to empty its contents) [28] and rate of store depletion during repetitive depolarization [15] reported previously in adult muscle fibers from dCasq-null mice. Therefore, we propose that the mechanism for faster I_SkCRAC_ activation observed in dCasq-null myotubes is a direct result of reduced SR Ca^2+^ buffering making it easier for repetitive depolarization (and RyR1 activation) to deplete junctional SR Ca^2+^ content to levels sufficient to open nearby SOCE channels. Consistent with this idea, total SR Ca^2+^ content is significantly reduced in Casq-deficient myotubes and muscle fibers [22,39].

Our finding that the maximal rate of I_SkCRAC_ activation is accelerated in dCasq-null myotubes are consistent with an increase in the maximal rate of Mn^2+^ quench of fura-2 fluorescence after caffeine/ryanodine-induced SR Ca^2+^ release in adult muscle fibers following siRNA-mediated knockdown of Casq1 [22]. However, Zhao et al [22] also reported that in spite of an increase in Mn^2+^ quench with Casq1 knockdown, expression of Orai1, the protein that forms the Ca^2+^ permeable SOCE channel, was reduced. On the other hand, our results indicate that maximal SOCE current density was not significantly altered in dCasq-null myotubes. These apparently divergent results could reflect differences in preparation (adult fibers vs. myotubes), transient/partial versus constitutive/complete Casq1-deficiency, or that Orai1 expression levels are not limiting for SOCE channel function in muscle. In addition, while our studies focus on changes in I_SkCRAC_, which is mediated by STIM1-Orai1 coupling [24], we cannot rule out potential contributions due to changes in other forms of Ca^2+^ entry (e.g. STIM1/TrpC channels).

### Azumolene does not directly inhibit I_SkCRAC_

Dantrolene, a skeletal muscle relaxant [40,41], is currently the only FDA-approved drug for the treatment of MH [1]. Dantrolene, and presumably its more water-soluble analogue azumolene, bind with high affinity to skeletal muscle SR membrane preparations, but not to sarcolemmal or t-tubule membranes [42,43]. A discrete dantrolene binding site within RyR1 (a.a. 590-609) was identified [43] and proposed to suppress RyR1 channel opening [44] by promoting RyR1 interdomain interactions that stabilize the closed state of the channel [45]. While dantrolene reduces electrically-evoked Ca^2+^ release in myotubes [46], as well as both SR Ca^2+^ release and [^3^H]ryanodine binding to SR vesicles [47], two separate studies failed to observe a direct effect of dantrolene on purified RyR1 channel gating following incorporation in artificial planar lipid bilayers [46,48]. An inhibitory effect of dantrolene on excitation-coupled calcium entry [46], likely due inhibition of L-type calcium channel activity [48,49], was demonstrated in both mouse myotubes and adult muscle fibers. In spite of these advances, the precise molecular target by which dantrolene acts to prevent and reverse an MH reaction remains controversial. 

The therapeutic action of dantrolene and azumolene in MH was recently suggested to result from an inhibition of SOCE coupled to RyR1 activity [21-23]. Using indirect fura-2 Ca^2+^ imaging and Mn^2+^ quench assays, these studies demonstrated that pretreatment with 20 µM azumolene for 1-2 min inhibited SOCE activated by subsequent addition of caffeine and ryanodine to promote RyR-dependent SR Ca^2+^ store depletion in mouse myotubes [23] and adult muscle fibers [22]. Interestingly, SOCE was not reduced when azumolene was added after caffeine and ryanodine application or when applied before thapsigargin-induced store depletion. Based on these results, the authors proposed that dantrolene and azumolene inhibit a specific component of SOCE that is linked to RyR1 channel activity. Under our experimental conditions, we activated SOCE channels in myotubes by repetitive depolarization-induced RyR1 Ca^2+^ release, while SR Ca^2+^ reuptake was inhibited by high concentrations of intracellular EGTA. While this approach activates SOCE linked to RyR1 channel activity [24], we found that neither I_SkCRAC_ magnitude nor rate of activation were significantly altered by pretreatment of WT and Y524S/+ myotubes with 50 µM azumolene. Similarly, application of 50 µM azumolene did not significantly alter I_SkCRAC_ current density even when applied after maximal I_SkCRAC_ activation (data not shown). These data indicate that azumolene is not a direct SOCE channel inhibitor. 

If SR Ca^2+^ store depletion and uncontrolled Ca^2+^ entry through SOCE channels is a key component of an MH reaction as suggested above, how could dantrolene (or azumolene) be highly effective in preventing and reversing MH crises if it does not act by blocking SOCE channels? As one possibility, since Orai1-dependent SOCE lies downstream of store depletion, anesthetic- or heat-induced RyR1-dependent Ca^2+^ leak would first lead to store depletion and only then be followed by SOCE activation, Ca^2+^ entry, and myoplasmic Ca^2+^ overload. According to this mechanism, MH events could be attenuated by agents that either reduce RyR1 Ca^2+^ leak and store depletion (e.g. dantrolene/azumolene) or directly inhibit Orai1-dependent Ca^2+^ influx when stores are depleted (e.g. SOCE channel inhibitors). Moreover, an agent that inhibits RyR1-mediated store depletion (e.g. dantrolene) would be expected to indirectly reduce SOCE activation even if the drug does not directly block the channel once it is activated. While our results demonstrate that azumolene does not directly inhibit Orai1 Ca^2+^ current, the drug could still indirectly limit I_SkCRAC_ activation by protecting stores from becoming depleted. In this context, the reduction in SOCE (i.e. Mn^2+^ quench) when azumolene was applied prior to caffeine/ryanodine exposure reported previously [21-23] could be the result of the drug reducing RyR1 Ca^2+^ release, and thus, limiting the relative degree of SR Ca^2+^ store depletion and SOCE activation achieved. 

## Conclusions

We used a previously described voltage clamp approach [24] to directly quantify the activation rate, magnitude, electrophysiological properties, and pharmacological signature of SOCE channel function (I_SkCRAC_) in myotubes derived from two separate mouse models for heat- and anesthetic-induced MH. We demonstrated a similar significant increase in the rate of I_SkCRAC_ activation during repetitive stimulation in myotubes derived from both models. While dantrolene and azumolene may minimize I_SkCRAC_ activation by reducing RyR1 Ca^2+^ release, and thus store depletion, the drugs do not to directly inhibit SOCE channels. Nevertheless, it will be important for future work to determine the potential therapeutic value of inhibiting SOCE channels in skeletal muscle in the prevention and management of MH environmental/exertional heat illness.

## Supporting Information

File S1 **Table S1.** Times for 10%, 50%, and 90% activation of I_SkCRAC_ at room temperature in C57Bl6, Y524S/+, and dCasq-null myotubes. **Table S2.** Times for 10%, 50%, and 90% activation of I_SkCRAC_ at physiological temperature in C57Bl6, Y524S/+, and dCasq-null myotubes. **Table S3.** Effect of azumolene (Azum, 50 µM) on times for 10%, 50%, and 90% activation of I_SkCRAC_ at in C57Bl6 and Y524S/+ myotubes. **Figure S1.** **I_SkCRAC_ current density and pharmacology.** (**A**) Average (±SE) I_SkCRAC_ current density recorded from WT C57Bl6 , Y524S/+, and dCasq-null myotubes at -80 mV in control (Cntl, *filled* *bars*) and after addition of 1 µM Gd^3+^ (*open bars*). Data for wild-type are taken from Yarotskyy and Dirksen (2012). Numbers of paired experiments are shown in bars. ^*^p < 0.05. (**B**) Average (±SE) I_SkCRAC_ current density recorded from WT C57Bl6, Y524S/+ , and dCasq-null myotubes at -80 mV in control (Cntl, black bars) and after addition of either 100 µM (C57Bl6 and Y524S/+) or 10 µM (dCasq-null) 2-APB (*open bars*). Numbers of paired experiments are shown in bars. ^*^p < 0.05. **Figure S2.** **The effect of PT on I_SkCRAC_ magnitude and activation rate in myotubes from WT C57Bl6, Y524S/+, and dCasq-null mice.** (**A**) Average (±SE) time required for 10% (T10%), 50% (T50%), and 90% (T90%) activation of I_SkCRAC_ in WT C57Bl6, Y524S/+, and dCasq-null myotubes. (**B**) Average (±SE) maximum rate of I_SkCRAC_ activation in WT C57Bl6, Y524S/+, and dCasq-null myotubes. ^*^p < 0.05. (**C**) Average (±SE) peak I_SkCRAC_ current density recorded at -80 mV in myotubes from WT, Y524S/+, and dCasq-null mice.
(DOCX)
