# Supplementary material for: Accelerated Activation of SOCE Current in Myotubes from Two Mouse Models of Anesthetic- and Heat-Induced Sudden Death
Source: PLoS One. 2013 Oct 15;8(10):e77633. doi: 10.1371/journal.pone.0077633 (PMC3797063; doi:10.1371/journal.pone.0077633)
Supplement: File S1 — Table S1. Times for 10%, 50%, and 90% activation of ISkCRAC at room temperature in C57Bl6, Y524S/+, and dCasq-null myotubes. Table S2. Times for 10%, 50%, and 90% activation of ISkCRAC at physiological temperature in C57Bl6, Y524S/+, and dCasq-null myotubes. Table S3. Effect of azumolene (Azum, 50 µM) on times for 10%, 50%, and 90% activation of ISkCRAC at in C57Bl6 and Y524S/+ myotubes. Figure S1. ISkCRAC current density and pharmacology. (A) Average (±SE) ISkCRAC current density recorded from WT C57Bl6 , Y524S/+, and dCasq-null myotubes at -80 mV in control (Cntl, filled bars) and after addition of 1 µM Gd3+ (open bars). Data for wild-type are taken from Yarotskyy and Dirksen (2012). Numbers of paired experiments are shown in bars. *p < 0.05. (B) Average (±SE) ISkCRAC current density recorded from WT C57Bl6, Y524S/+ , and dCasq-null myotubes at -80 mV in control (Cntl, black bars) and after addition of either 100 µM (C57Bl6 and Y524S/+) or 10 µM (dCasq-null) 2-APB (open bars). Numbers of paired experiments are shown in bars. *p < 0.05. Figure S2. The effect of PT on ISkCRAC magnitude and activation rate in myotubes from WT C57Bl6, Y524S/+, and dCasq-null mice. (A) Average (±SE) time required for 10% (T10%), 50% (T50%), and 90% (T90%) activation of ISkCRAC in WT C57Bl6, Y524S/+, and dCasq-null myotubes. (B) Average (±SE) maximum rate of ISkCRAC activation in WT C57Bl6, Y524S/+, and dCasq-null myotubes. *p < 0.05. (C) Average (±SE) peak ISkCRAC current density recorded at -80 mV in myotubes from WT, Y524S/+, and dCasq-null mice. (DOCX) [file pone.0077633.s001.docx]

**Table S1.**

| **Type of Cell** | **T10% (s)** | **T50% (s)** | **T90% (s)** | **n** |
| --- | --- | --- | --- | --- |
| C57Bl6 | 6.9 ± 0.9 | 18.6 ± 2.0 | 44.9 ± 4.8 | 28 |
| Y524S/+ | 3.7 ± 0.6^*^ | 11.6 ± 1.1^*^ | 33.8 ± 2.2 | 21 |
| dCasq-null | 3.8 ± 0.5^*^ | 11.1 ± 1.1^*^ | 29.8 ± 3.6^*^ | 25 |

**, p < 0.05 between WT and either dCasq-null or Y524S/+ groups*

**Table S2.**

| **Type of Cell** | **T10% (s)** | **T50% (s)** | **T90% (s)** | **n** |
| --- | --- | --- | --- | --- |
| C57Bl6^#^ | 1.0 ± 0.2 | 6.4 ± 2.0 | 22.4 ± 7.5 | 11 |
| Y524S/+ | 0.7 ± 0.1 | 3.8 ± 0.3 | 9.8 ± 1.2 | 7 |
| dCasq-null | 0.6 ± 0.1 | 2.8 ± 0.3 | 8.5 ± 1.3 | 11 |

***^#^****Data taken from Yarotskyy and Dirksen, 2012 (*[*24*](#_ENREF_24)*).*

| **Table S3. Type of Cell** | **T10% (s)** | | **T50% (s)** | **T90% (s)** | **n** |
| --- | --- | --- | --- | --- | --- |
| C57Bl6 | | 7.1 ± 1.1 | 18.8 ± 1.7 | 47.7 ± 2.9 | 6 |
| C57Bl6 + Azum | | 11.2 ± 2.4 | 27.3 ± 5.5 | 55.4 ± 10.5 | 6 |
| Y524S/+ | | 5.0 ± 1.7 | 13.1 ± 2.5 | 34.8 ± 5.4 | 6 |
| Y524S/+ + Azum | | 6.2 ± 0.4 | 17.5 ± 1.1 | 42.7 ± 4.5 | 8 |


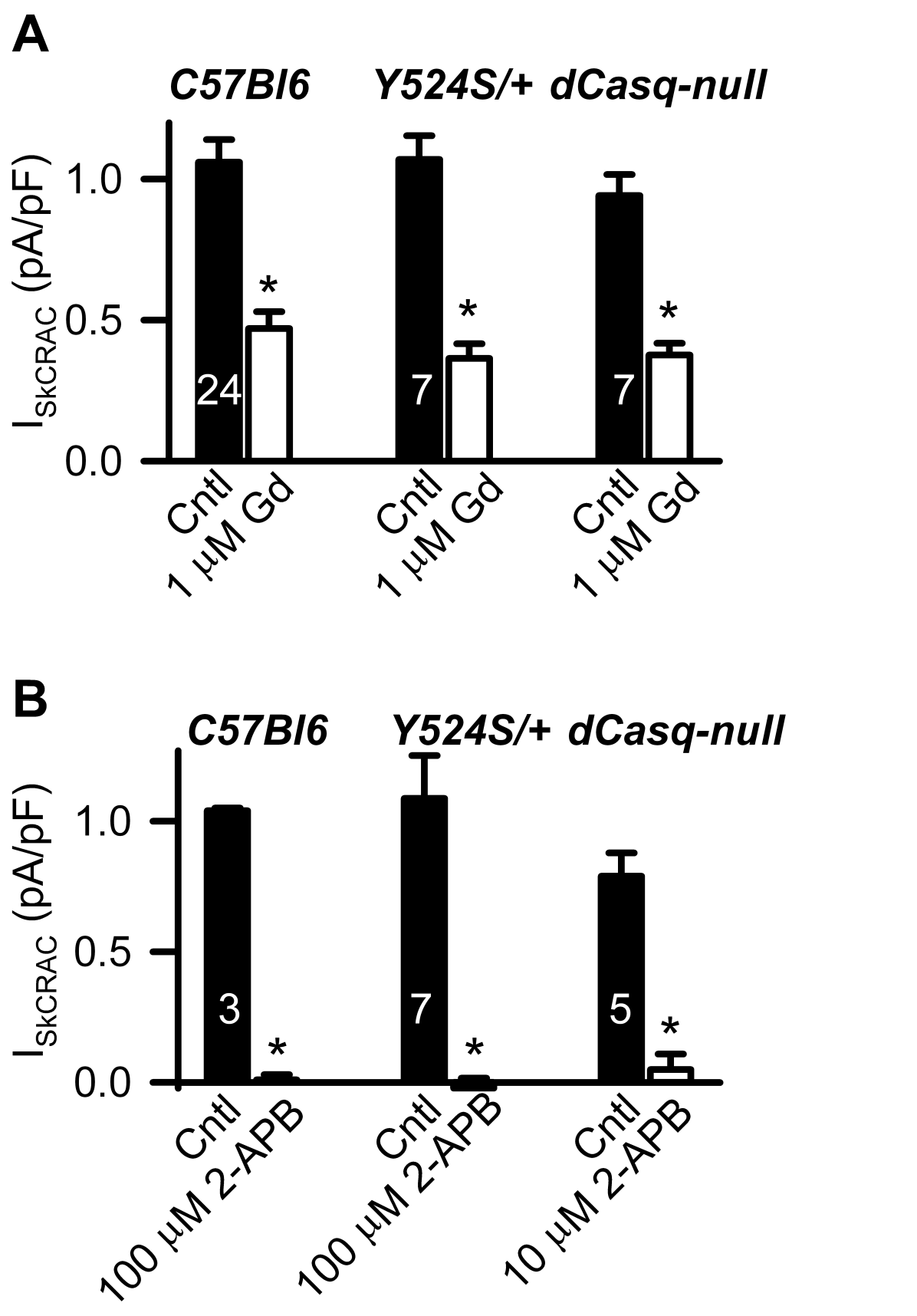


**Figure S1.**

**
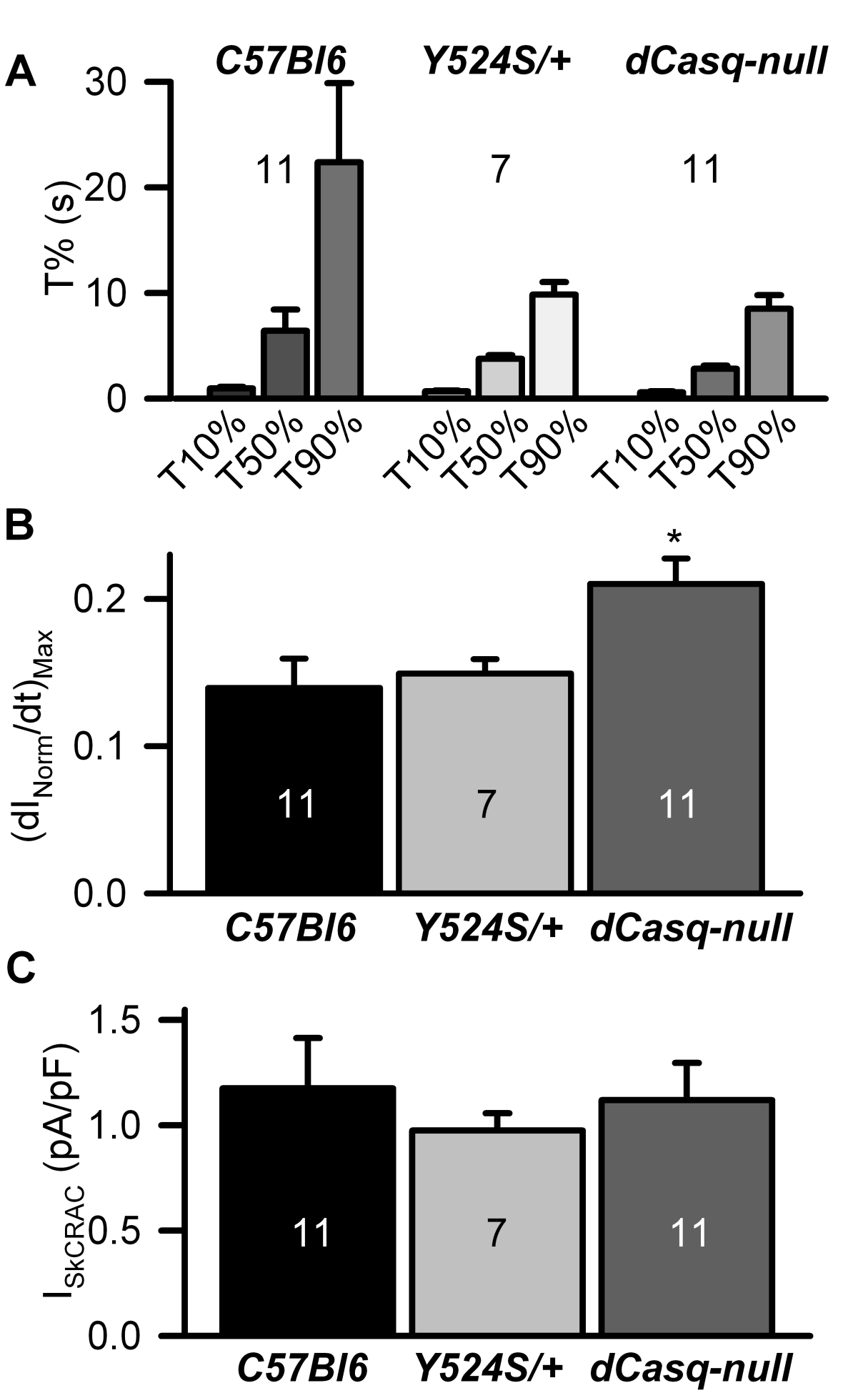
**

**Figure S2.**
